# Computer-Aided Telephone Support for Primary Care Patients with Common Mental Health Conditions: Randomized Controlled Trial

**DOI:** 10.2196/10224

**Published:** 2018-12-10

**Authors:** Salaha Zaheer, Vanessa Garofalo, David Rodie, Athina Perivolaris, Jenny Chum, Allison Crawford, Rose Geist, Andrea Levinson, Brian Mitchell, David Oslin, Nadiya Sunderji, Benoit H Mulsant, PARTNERs Study Group

**Affiliations:** 1 Geriatric Mental Health Services Centre for Addiction and Mental Health Toronto, ON Canada; 2 Centre for Addiction and Mental Health Toronto, ON Canada; 3 Department of Psychiatry University of Toronto Toronto, ON, ON Canada; 4 Trillium Health Partners Mississauga, ON Canada; 5 Group Health Centre Sault Ste Marie, ON Canada; 6 University of Pennsylvania and the Department of Veteran Affairs Philadelphia, PA United States; 7 St Michael's Hospital Toronto, ON Canada

**Keywords:** telemedicine, collaborative care, depression, anxiety, at-risk drinking, lay provider, family medicine, general practice, primary care psychiatry

## Abstract

**Background:**

Depression, anxiety, and at-risk drinking are highly prevalent in primary care settings. Many jurisdictions experience geographical barriers to accessing mental health services, necessitating the development and validation of alternative models of care delivery. Existing evidence supports the acceptability and effectiveness of providing mental health care by telephone.

**Objective:**

This analysis assesses patient’s acceptability of computer-aided telephone support delivered by lay providers to primary care patients with depression, anxiety, or at-risk drinking.

**Methods:**

The Primary care Assessment and Research of a Telephone intervention for Neuropsychiatric conditions with Education and Resources study is a randomized controlled trial comparing a computer-aided telephone-based intervention to usual care enhanced by periodic assessments in adult primary care patients referred for the treatment of depression, anxiety, or at-risk drinking; no part of the study involves in-person contact. For this analysis, the following data were obtained: reasons provided for declining consent; reasons provided for withdrawing from the study; study retention rate; and a thematic analysis of a satisfaction survey upon study completion.

**Results:**

During the consent process, 17.1% (114/667) patients referred to the study declined to participate and 57.0% of them (65/114) attributed their refusal to research-related factors (ie, randomization and time commitment); a further 16.7% (19/114) declined owing to the telephone delivery of the intervention. Among the 377 participants who were randomized to the 1-year intervention, the overall retention rate was 82.8% (312/377). Almost no participants who withdrew from the study identified the telephone components of the study as their reason for withdrawal. Analysis of a qualitative satisfaction survey revealed that 97% (38/39) of comments related to the telephone components were positive with key reported positive attributes being accessibility, convenience, and privacy.

**Conclusions:**

Our results suggest that a computer-aided telephone support is highly acceptable to primary care patients with depression, anxiety, or at-risk drinking. In particular, these patients appreciate its accessibility, flexibility, and privacy.

**Trial Registration:**

ClinicalTrials.gov NCT02345122; https://clinicaltrials.gov/ct2/show/NCT02345122 (Archived by WebCite at http://www.webcitation.org/73R9Q2cle)

## Introduction

### Background

In Canada, 1 in 5 individuals report experiencing symptoms of substance abuse and mental health problems each year [1,2], and almost 75% of mental health visits are related to mood and anxiety disorders [3].The economic impact of depression alone is estimated to be over Can $32 billion, which is twice the amount of money spent on mental health and community care [4]. In Ontario, 15% of adults have consulted a family physician or a psychiatrist about their mental health in the past year, and mental health visits represent 10% of all physician visits [5]. More of these visits occur in the primary care settings than in the psychiatric setting [5,6]. Despite the increased focus on mental health, an estimated 1.6 million Canadian citizens report that their needs for mental health were unmet with 36% reporting that their needs for counseling services were either unmet or partially met [7]. In a recent survey, wait times to see a psychiatrist ranged from 15 to 59 weeks, and wait times to start psychotherapy ranged from 3 to 22 weeks [8].

### Prior Work and Rationale

With long wait times and unmet needs for mental health service, alternative approaches to service delivery have been proposed and evaluated. A literature search was conducted to identify relevant examples of studies that investigated the feasibility and impact of using lay coaches to provide mental health management and support over the phone [9-18]. These studies conducted in the United States or Canada used telephone coaching to provide a range of interventions as follows: self-help resources, symptom tracking, promotion of behavioral activation and self-management, or treatment adherence. Their main findings are summarized in Table 1. Collectively, these studies suggest the acceptability and efficacy of offering support and care via telephone to primary care patients with depression, anxiety, or at-risk drinking. These studies were identified with PubMed using the following keywords: “lay coach,” “telephone support,” “mental health,” “depression,” “anxiety,” and “alcohol use.” We included studies that were judged to be most relevant and met the following criteria: use of a telephone component; use of a lay coach; and focus on depression, anxiety, or alcohol use. Not all interventions described in these studies were successful for all patients. Thus, we are conducting a study to assess the feasibility and impact of a computer-aided telephone-based intervention for primary care patients with depression, anxiety, or at-risk drinking: the Primary care Assessment and Research of a Telephone intervention for Neuropsychiatric conditions with Education and Resources study (PARTNERs; ClinicalTrials.gov Identifier: NCT02345122). PARTNERs utilizes Mental Health Technicians (MHT; coaches) who provide mental health support to patients over the telephone with the help of standardized questionnaires and assessment reports available on the Behavioral Health Laboratory (BHL) software (Capital Solutions, PA, USA). This paper evaluates the acceptability of this intervention and potential limitations from the patient’s perspective.

**Table 1:** Summary of most relevant published studies of telephone-based support for depression, anxiety, or at-risk drinking.

Study and location	Study design	Main findings
Simon et al (2000) [9], United States (n=613)	Patients starting antidepressant trial randomized to 3 groups: (1) usual care; (2) telephone contacts every 3 months with feedback only; and (3) telephone contacts every 3 months with feedback and support.	Patients who received telephone feedback and support were more likely to receive an adequate antidepressant dosage; have lower depression scores; and have a lower likelihood of persistent major depression. Feedback only had no significant effect on the outcomes.
Oslin et al (2003) [10], United States (n=97)	Veteran participants (n=97) with depression and at-risk drinking were assigned to 2 groups: (1) usual care and (2) TDM^a^ by a behavioral health specialist. Patients in the TDM received regular follow-ups for 24 wk. Symptomatic outcomes were assessed at 4-months.	TDM was associated with improved outcomes for depression and at-risk drinking: response rates were 39% in the TDM group and 18% in the usual care group.
Brown et al (2007) [11], United States (n=819)	12-month randomized comparison of a telephone intervention and a mail intervention for primary care patients (n=819) with alcohol use disorders. Participants received telephone counseling (motivational interviewing) or pamphlets on healthy lifestyle. Drinking levels were measured after 3 months.	Larger reduction in alcohol consumption was observed in the telephone group than in the mail group (males: 17.3% vs 12.9%; females: 13.9% vs 11.0%) The number of telephone counseling sessions was associated with the reduction in drinking.
McCusker et al (2012) and Simco et al (2015) [12,17], Canada (n=63)	Open, noncontrolled design. Participants with comorbid depression and chronic physical illness received self-care tools and telephone support by a lay coach for 6 months.	The telephone intervention was found to be feasible and acceptable: 91% (57/63) of the participants completed the 2-month follow-up; 63% (mean 5.7/9) of possible calls were completed. Participants experienced significant improvement in depression symptoms at 6 months.
Mello et al (2013) [13], United States (n=285)	Injured adults screening positive for alcohol use and discharged from an emergency room randomized to 2-call phone intervention or usual care. Outcomes were measured after 12 months.	Alcohol-related injuries were lower in the phone intervention group with no difference in consumption and other alcohol-related consequences.
Pickett et al (2014) [14], United States (n=124)	12-wk randomized trial of telephone-facilitated depression care and usual care in recently discharged primary care patients.	No significant difference in outcomes between facilitated and routine care.
McCusker et al (2015) and McClusker et al (2017) [15,16] (n=223)	Randomized trial of a depression self-care tool kit, with and without telephone coaching in primary care adults with depression and comorbid chronic physical condition. Outcomes were measured after 3 and 6 months.	77.1% completed the 6-month assessment. PHQ-9^b^ scores were significantly different after 3 months but not after 6 months. The benefit of coaching on 6-month PHQ-9 was seen only among participants who were not receiving baseline psychological treatment. No significant differences in secondary outcomes (self-efficacy, satisfaction, and use of health services).
Rollman et al (2017) [18], United States (n=329)	Patients with anxiety randomized to a telephone-delivered CC^c^ intervention or usual-care referral. Participants in the CC group received help from a nonmental health professional for 12 months.	Patients randomized to CC had improved mental health-related quality of life, anxiety symptoms, and mood at the 12-month follow-up compared with usual care.

^a^TDM: telephone disease management.

^b^PHQ-9: Patient Health Questionnaire-9 [19].

^c^CC: collaborative care.

## Methods

### Setting

PARTNERs is a randomized controlled trial that aims to assess the feasibility and impact of computer-aided telephone monitoring and support for primary care patients with depression, anxiety, or at-risk drinking using an integrated care model. As of April 30, 2017, the project has been implemented at 18 primary care sites, comprising 189 primary care providers (PCPs; ie, family physicians and nurse practitioners) in urban, suburban, and rural settings across Ontario.

### Participants Eligibility and Recruitment

Starting in November 2014, PCPs identified adult patients with symptoms of depression, anxiety, or at-risk drinking; obtained their verbal permission to refer them to the study; and completed a brief referral form including the patient’s phone number and preferred time of contact. Research associates (RAs) called these patients within 5 business days and obtained their consent to participate via phone, following a process approved by the Research Ethics Board of the Centre for Addiction and Mental Health (CAMH). Starting with this call and at the beginning of each call, participants were asked to confirm their date of birth to verify their identity. Participants were then scheduled for a baseline assessment to confirm that they met all the inclusion criteria (receiving care from a PCP; referred to the study by their PCP because of depression, anxiety, or at-risk drinking; age 18 years and older; access to a telephone; willingness and ability to converse in English by telephone; willingness and ability to provide informed consent). Participants were excluded if they met one of the following exclusion criteria: psychotic disorder; bipolar disorder; obsessive-compulsive disorder; post-traumatic stress disorder; current substance use disorder except for alcohol use disorder; cognitive impairment as defined by a score of 16 or higher on the Blessed Orientation Memory Concentration test [20]; high risk for suicide; physical condition requiring hospitalization; or expected to die during the next 6 months.

### Assessments

Participants were called by an RA at baseline and after 4, 8, and 12 months and completed a comprehensive assessment using the BHL software. Additional data were obtained regarding reasons for declining to participate in the study, reasons for withdrawing from the study, and satisfaction with participation. Patients who declined consent were asked for their reason(s) and their answers were recorded in a tracking log. Patients who consented but subsequently withdrew before completing the baseline assessment were also asked for their reason(s). When participants withdrew later during the study, reasons were similarly obtained and recorded.

During the 12-month follow-up assessment, participants completed a satisfaction survey including 5 open-ended questions (“Do you have any comments about access or entry to services?”; “Please comment on aspects of your experience with this treatment or support service that were particularly helpful to you”; “Please comment on aspects of your experience with this treatment or support service that you feel could be improved”; and “Any additional comments?”) and one 4-point global rating of the services provided (ie, poor, fair, good, and very good). The satisfaction survey was completed by telephone using a REDCap (Vanderbilt University, Nashville, Tennessee with ongoing support from the US National Institutes of Health) database.

### Intervention

After completing the baseline assessment, eligible participants were randomized to either usual care plus research assessments and telephone support (“the intervention”) or usual care enhanced by the research assessments (“enhanced usual care”).

Using electronic faxes, PCPs were provided with the results of the 4 research assessments for all participants and were contacted as needed clinically (eg, if a participant reported some suicidality). In addition, participants randomized to the intervention received telephone calls from an MHT; typically, these phone calls took place weekly at the initiation of the intervention and tapered off to monthly as participants improved. This decision was based on remission of symptoms as defined by a score of <10 on Patient Health Questionnaire-9 or a decrease of 50% compared with the baseline score: after remission was maintained for at least 1 month, the frequency of the calls was reduced from weekly to biweekly; after remission was maintained for at least another month, the frequency of the calls was decreased to monthly. The first phone call lasted about 1 hour and the subsequent phone calls lasted 20-30 minutes; all calls were scheduled at times convenient to the participant, including evenings but not weekends. MHTs were bachelor-level trained lay providers. Their main role was to support participants’ self-management by monitoring symptoms and treatment adherence, providing education on contributory lifestyle factors, facilitating healthy lifestyle, and communicating updates and recommendations to their PCP [21]. MHTs also facilitated goal setting using a stages-of-change model and motivational interviewing techniques to set Specific; Measurable; Attainable; Relevant; Timely goals. MHTs received weekly supervision from the project psychiatrist.

### Data Analysis

This analysis is based on all data collected until April 30, 2017. Descriptive statistics characterize the participants.

For this analysis, the main measures of acceptability of the telephone-based intervention were as follows: the proportion of referred patients who declined to consent or withdrew before completing the baseline and identified telephone services as their reason for doing so and the overall retention rate. In addition, a content analysis of the qualitative information in the consent tracking log and the satisfaction survey was conducted to characterize the intervention acceptability. Reasons for declined consent and withdrawal prior to completing the baseline were combined. Responses to the overall satisfaction rating and the 4 open-ended questions from the satisfaction survey were analyzed; responses that included comments related to the telephone component of the project were categorized and counted.

## Results

### Flow and Characteristics of Participants

**Figure 1:**
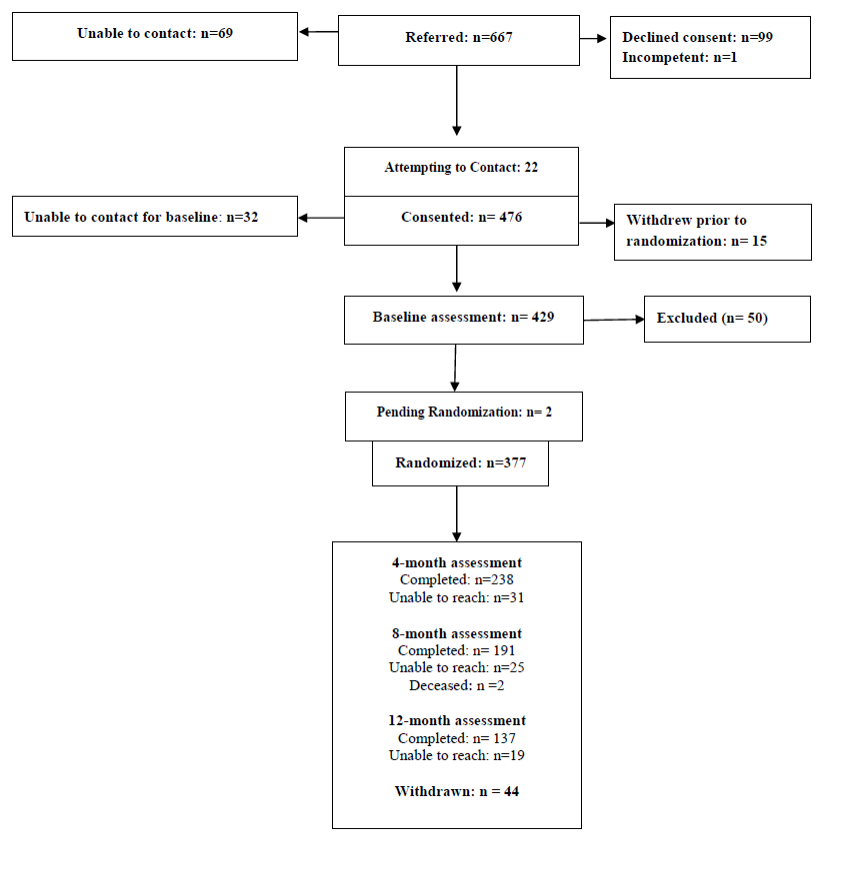
Flow of participants (November 1, 2014 to April 30, 2017).

Figure 1 summarizes the flow of the 667 patients who were referred to the study; of these, 10.3% (69/667) could not be contacted, 0.1% (1/667) had been deemed incompetent to consent; 14.8% (99/667) declined to consent, and an additional 2.2% (15/667) consented but withdrew before completing the baseline. Moreover, 4.8% (32/667) could not be contacted to complete the baseline assessment, resulting in 64.3% (429/667) who completed the baseline assessment. The demographic and clinical characteristics of these 429 participants are presented in Table 2. Of the participants who completed their baseline assessment, 87.9% (377/429) were randomized, of whom 2 died, 44 withdrew before completing the study, and 19 could not be reached for their 12-month assessment, yielding an overall retention rate of 82.8% (312/377).

### Reasons Provided for Declining Participation in the Study

Of the participants who declined consent (n=99) or withdrew prior to the baseline assessment (n=15), 15 did not provide any reasons for their refusal. Of the 121 reasons provided by the other participants that are presented in Table 3, only 15.7% (19/121) were explicitly related to concerns with the telephone component of the project or to a preference to see a therapist in person.

### Reasons Provided for Withdrawing from the Study

Of the 44 participants who withdrew from the study after being randomized, 10 did not provide a reason for their withdrawal. The 41 reasons provided by other participants are shown in Table 4; none were attributed to the telephone intervention.

### Satisfaction Survey

**Figure 2:**
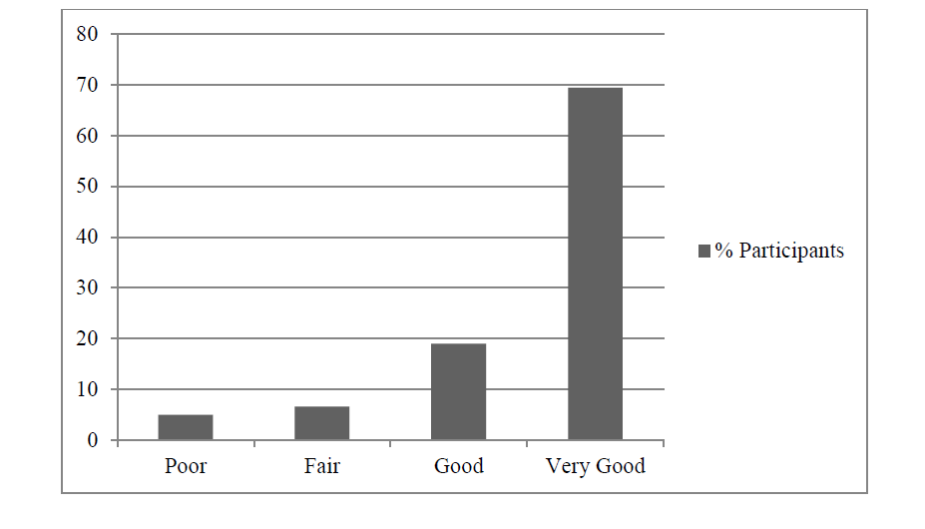
Distribution of responses to the question “overall, how would you rate the services you received?”(n=121).

The overall satisfaction ratings are presented in Figure 2. In open-ended responses, 39 participants made 45 comments on the use of the telephone in the study (Table 5). Moreover, 16% (7/45) of these comments were negative and 84% (38/45) were positive, emphasizing the accessibility and convenience of telephone calls (ie, being able to speak with someone from their home) or the privacy and relative anonymity of the calls.

**Table 2:** Characteristics of the 429 participants who completed the baseline assessment.

Characteristic		Value
**Age (years)**		
	Mean (SD)	41.7 (15.8)
	Median (range)	38 (18-90)
	Q1-Q3	29-54
**Sex, n (%)**		
	Female	294 (68.5)
**Ethnicity, n (%)**		
	White	344 (80.2)
	Asian/Pacific Islander	29 (6.8)
	Native Canadian	16 (3.7)
	Black/African Canadian	15 (3.5)
	Other/Mixed	25 (5.8)
**Self-reported general health, n (%)**		
	Excellent	24 (5.6)
	Very good	86 (20.0)
	Good	188 (43.8)
	Fair	98 (22.8)
	Poor	33 (7.7)
**Education, n (%)**		
	Less than high school	35 (8.2)
	High school graduation	88 (20.5)
	Some college or university	163 (38.0)
	University degree	102 (23.8)
	Postgraduate degree	34 (7.9)
	Other	7 (1.6)
**Employment, n (%)**		
	Full-time	162 (37.8)
	Part-time	63 (14.7)
	Not working	204 (47.6)
**Marital status, n (%)**		
	Married or partnered	186 (43.4)
	Never married	166 (38.7)
	Divorced or separated	57 (13.2)
	Widowed	20 (4.7)
**Patient Health Questionnaire-9**		
	Mean (SD)	14.0 (6.0)
	Minimal (0-4), n (%)	19 (4.4)
	Mild (5-9), n (%)	84 (19.6)
	Moderate (10-14), n (%)	130 (30.3)
	Moderately severe (15-19), n (%)	106 (24.7)
	Severe (20-27), n (%)	90 (21.0)
**Generalized anxiety disorder**		
	Mean (SD)	11.6 (5.5)
	Minimal (0-4), n (%)	45 (10.5)
	Mild (5-9), n (%)	115 (26.8)
	Moderate (10-14), n (%)	130 (30.3)
	Severe (15-21), n (%)	139 (32.4)
**Alcohol use**		
	At-risk drinker^a^	156 (36.4)
	Number of weekly standard drinks, mean (SD)	7.2 (12.8)
	Median (range)	2 (0-113)
	Q1-Q3	0-9

^a^At-risk drinker: males with 15 or more drinks per week or 5 or more in a given day; females with 10 or more drinks per week or 4 in a given day; or participants endorsing 2 or more symptoms of The Diagnostic and Statistical Manual of Mental Disorders, Fifth Edition Alcohol Use Disorder.

**Table 3:** Reasons given for declining consent or withdrawing prior to completing the baseline assessment.

Reasons			Number of times reason was given (n=121)^a^, n (%)
Concerns with telephone components or prefers in-person assessment and treatment			19 (15.7)
**Concerns with other research components**			65 (53.7)
	Time commitment (30)^b^		N/A^c^
	Not a good fit (11)^b^		N/A
	Privacy concerns (9)^b^		N/A
	Concerns with research participation or design (8)		N/A
	**Communication and logistic barriers (7)^b^**		
		Moving (4)^b^	N/A
		Language or hearing problems (2)^b^	N/A
		Unavailable during study times (1)^b^	N/A
Prefers or already pursuing other treatment			18 (14.9)
**Does not believe treatment is needed**			15 (12.4)
	Feeling better (14)^b^		N/A
	Not interested in seeking help (1)^b^		N/A
Other reasons			4 (3.3)

^a^121 reasons provided by 99 patients who declined consent and 15 who withdrew prior to completing the baseline assessment (some provided multiple responses).

^b^The number of patients who provided “time commitment” as the reason.

^c^N/A: not applicable.

**Table 4:** Reasons given for withdrawal after randomization.

Reasons	Number of times reason given (n=41)^a^, n (%)
Study not helpful	19 (46)
Time commitment	9 (22)
Uncomfortable with assessments	3 (7)
Prefers pursuing other treatment	3 (7)
Feeling better	3 (7)
Expected counseling	2 (5)
Other reasons	2 (5)

^a^41 reasons provided by 44 participants who withdrew after randomization (some provided multiple reasons; some provided no reasons).

**Table 5:** Major topics related to use of phone from the satisfaction survey.

Topics		Number of comments (n=45)^a^, n (%)
**Positive comments**		38 (84)
	Accessibility and convenience of calls	21 (47)
	Flexibility	7 (16)
	Privacy and anonymity	4 (9)
	Liked phone calls	4 (9)
	Text reminders	2 (4)
**Negative comments**		7 (16)
	Would prefer in-person services	4 (9)
	Barriers associated with telephone use	3 (7)

^a^45 comments provided by 39 participants (some provided multiple comments).

## Discussion

### Principal Findings

We assessed the acceptability of computer-aided telephone assessments and support for primary care patients with depression, anxiety, and at-risk drinking. Telephone services were highly acceptable, as demonstrated by high consent and retention rates and overall positive feedback. Only a small proportion of referred patients cited the use of telephone as their main reason for declining to participate and none cited this as a reason for withdrawing after randomization.

### Comparisons with Prior Work

Our results are congruent with those of several previous studies that have demonstrated telephone as an effective means of engaging some patients in mental health screening and interventions [9-13,18]. Several of our findings deserve further comment. First, we were unable to reach and engage approximately 10.3% (69/667) of the patients referred to the study despite attempting to call them up to 10 times over a period of 1 month. Similarly, despite multiple attempts, we could not complete the baseline assessment in 6.7% (32/476) of the participants who consented. Although these rates are low, they illustrate the decreased engagement opportunities of a telephone intervention compared with an intervention embedded in a practice setting. Also, although PCPs were informed about this inability to contact their patients, we do not know what happened to these patients.

Only a small proportion of those who were contacted declined to participate. The main reason cited was the time commitment required to participate in the study. The second reason was a preference for other treatment, typically counseling or psychotherapy. By contrast, only a few specifically mentioned being uncomfortable with telephone assessments and intervention. A few patients also explicitly indicated a preference for speaking with a trained professional. Thus, although lay providers may facilitate access to mental health care by increasing the supply of providers and decreasing costs, they may not be accepted by all patients.

The retention rate was over 80%, higher than the retention rate in most 12-month or shorter randomized studies of mental health interventions [22]. Previous studies of telephone interventions have shown similar high retention rates [9,12,14-17]. The use of cellular phones by almost all participants, in combination with appointment text reminders, may have contributed to the high retention rate because it facilitated participants’ availability. Taken together, these results support the acceptability of computer-aided telephone-based mental health support in primary care. Furthermore, our high retention rate in a study in which half of the participants were randomized to a low intensity condition (ie, telephone assessments every 4 months) suggests that frequent calls may not be needed to promote retention. In some prior studies, the retention rate was negatively correlated with the length of the study (as would be expected) and with the frequency of contacts [9,12,14,15,17]. This suggests that many patients prefer a shorter time commitment. After randomization, none of the relatively small number of participants who withdrew cited the use of telephone as their reason. The main reported reason was that “participation was not helpful,” but about one-fifth did not provide a reason for withdrawal. We did not identify specific characteristics (eg, age, gender, mental health condition) associated with withdrawal from the study (data not shown).

Finally, the satisfaction survey responses were almost universally positive and highlighted several advantages of a telephone intervention. As expected, participants identified accessibility and convenience. Access is particularly important in rural areas where resources are scarce [9]. A telephone intervention can also be used to engage those people for whom driving or other aspects of mobility are issues, such as older adults [9]. Participants also appreciated being contacted promptly and the flexible call times, obviating the need to take time off work or school. Some participants also identified privacy and anonymity as advantages of the telephone intervention. Thus, we believe it helped alleviate the stigma that remains attached to accessing mental health services. Similarly, some participants reported that the relative anonymity of telephone calls made it easier to disclose and discuss sensitive issues such as suicidal ideation, self-harm, or past traumas not previously disclosed to their PCP.

### Limitations

The main limitations are owing to our study not being designed to directly assess the acceptability of the phone intervention. First, some patients declined the referral to the study, and we did not collect the number of, or reasons for, these refusals. Though we believe that most were owing to concerns about participating in a randomized trial, some may have been because of the telephone intervention. Thus, our results may overestimate the acceptability of this type of service. A different study eliciting preference for an in person versus a telephone intervention, followed by randomization to one of these interventions, would be needed to compare the acceptability and adherence to these 2 types of interventions in the general patient population. However, the high retention rate supports the acceptability of the telephone intervention in those who consented to the study. Second, the satisfaction survey was completed during the last assessment and it is possible that we would have obtained less positive feedback from the small number of participants who discontinued the study early.

### Conclusion

Many patients in primary care settings cannot access traditional mental health care. Fully automated interventions (eg, Web-based therapy) offer potential innovative and cost-effective solutions to this problem [23,24]. Although these more advanced technologies are being developed, “plain-old telephone” can be combined with computer-based assessments and support. This approach seems to be highly acceptable to a large number of primary care patients. Furthermore, even when in-person or fully automated services are available, computer-aided telephone-based mental health services may have unique advantages for some subgroups of patients. We envision a future mental health system that optimizes access and quality by integrating multiple modes of service delivery—in person, by phone, and via Web-based and mobile platforms.

## Abbreviations

BHL: Behavioral Health Laboratory
CAMH: Centre for Addiction and Mental Health
MHT: Mental Health Technicians
NIH: National Institute of Health
PARTNERs: the Primary care Assessment and Research of a Telephone intervention for Neuropsychiatric conditions with Education and Resources study
PCP: primary care provider
RA: research associate

## Appendix

Multimedia Appendix 1 CONSORT-EHEALTH checklist (V 1.6.1).
